# Synergistic Effects of Nutrients on Musculoskeletal Health in Gerontology: Understanding the Combined Impact of Macronutrients and Micronutrients

**DOI:** 10.3390/nu16111640

**Published:** 2024-05-27

**Authors:** Yupeng Liu, Keyu Qian, Xiaodong Shi, Yuqi Jing, Hangqian He, Yiling Li, Dapeng Li, Shuran Wang

**Affiliations:** School of Public Health, Wenzhou Medical University, Wenzhou 325035, China; liuyupeng@wmu.edu.cn (Y.L.); qiankeyu2022@163.com (K.Q.); xiaodongshi1998@163.com (X.S.); jyq18091352188@163.com (Y.J.); hangqianhe@163.com (H.H.);

**Keywords:** osteoporosis, sarcopenia, musculoskeletal, aging, nutrition

## Abstract

With the global aging population, addressing prevalent age-related conditions such as osteoporosis and sarcopenia is crucial. Traditional nutritional strategies focusing on single nutrients like calcium, vitamin D, or protein have limitations, prompting a nuanced exploration of the relationship between aging, nutrition, and musculoskeletal health. This cross-sectional study examines the complex interplay between dietary intake of macronutrients, common micronutrients, and water, as well as their association with musculoskeletal health in adults aged 50 to 80 years, using U.S. National Health and Nutrition Examination Survey data (NHANES). Employing multiple linear regression, restricted cubic splines, weighted quantile sum (WQS), and quantile-based g-computation (QGC) regression models, our initial analysis using the WQS model revealed that a one-quartile increase in mixed macronutrient intake was associated with a significant 0.009 unit increase in bone mineral density (BMD) and a 0.670 unit increase in grip strength, while a similar increase in mixed micronutrient intake showed a 0.007 unit increase in BMD and a 0.442 unit increase in grip strength. Our findings highlight the importance of a balanced dietary approach in promoting musculoskeletal health in the elderly, offering holistic strategies for overall well-being.

## 1. Introduction

With the aging of the global population, age-related comorbidities increasingly affect the daily lives and well-being of older adults. Musculoskeletal disorders, which are particularly prevalent in this age group, have profound implications [1]. Osteoporosis and sarcopenia, two notable conditions, significantly compromise the health and quality of life of older adults [2].

Characterized by diminished bone mineral density (BMD) and microstructural deterioration, osteoporosis is a widespread chronic condition in the elderly population. These changes result in increased bone fragility and a heightened risk of fractures, making it a major concern for older adults [3]. Despite the widespread use of calcium and vitamin D (VD) supplementation in recent years, several studies have concluded that augmenting calcium intake among older individuals is unlikely to result in clinically significant reductions in fractures or progressive increases in bone mass [4,5].

Sarcopenia, which manifests as a progressive loss of skeletal muscle mass, strength, and function, presents its own set of challenges [6]. Handgrip strength, a measure of muscle strength, serves as a clinical indicator of mobility and is considered a more reliable predictor of clinical outcomes than low muscle mass alone [7]. While protein supplementation is a common intervention [8,9,10], current research suggests that its efficacy in combating sarcopenia is unsatisfactory.

Both macronutrients and micronutrients are critical for maintaining bone and muscle homeostasis. While numerous studies have examined the effects of individual nutrients on bone and muscle health, a singular focus on isolated key nutrient intake fails to consider the synergistic effects of overall dietary nutrition [11,12]. Therefore, it is imperative to conduct a holistic analysis. This requires a comprehensive analysis of the interplay between the diet—including energy, macronutrients, and micronutrients—and the onset of osteoporosis and muscle loss.

As individuals age, their basal metabolic rate decreases, lipid metabolism capability diminishes, digestive functions decline, muscles gradually atrophy, and overall hydration decreases. Concurrently, various tissues and organs undergo atrophy, resulting in reduced secretion of immune cells and a decline in immune function. This physiological decline often leads to a widespread deficiency in a variety of nutrients [1]. This deficiency, particularly in dietary energy and essential nutrients, is closely linked to the development of osteoporosis and muscle loss. Research has consistently shown that older adults are prone to energy deficiency, which in turn increases the risk of multiple nutrient deficiencies [13,14]. In light of these findings, it is imperative that the relationship between osteoporosis, muscle loss, and nutrition be examined from a holistic perspective. This approach should include a comprehensive assessment of dietary intake, including energy, macronutrients, and micronutrients, to fully understand and address the nutritional challenges faced by the aging population. To address these challenges, this study utilizes National Health and Nutrition Examination Survey (NHANES) data to explore the associations of mixed macronutrients and micronutrients intake with BMD and grip strength among older adults.

## 2. Materials and Methods

### 2.1. Study Population

We used data from the NHANES, a robust cross-sectional survey conducted by the Health Statistics Center of the American Centers for Disease Control and Prevention among non-institutionalized residents of the United States. The NHANES uses a carefully stratified and multistage sampling design to ensure the representation of diverse demographics in its samples. Baseline health and nutrition data were gathered through in-person interviews, comprehensive mobile physical examinations, and laboratory tests to ensure the accuracy and reliability of the data. Ethical considerations were paramount, and the NHANES protocol received formal approval from the Ethics Review Committee of the American National Health Statistics Center. Informed consent was obtained from each participant, underscoring the commitment to ethical research practices.

The selection of our study population was supported by an approach that took into account the specific dietary intake profiles we needed, covering a wide range of macronutrients and micronutrients. Additionally, we included femoral neck BMD measurements from the 2007–2008, 2009–2010, 2013–2014, 2017–2018, and 2019–2020 survey cycles. The dataset consisting of these five survey cycles is referred to as the bone mass dataset. To provide a comprehensive assessment of participants’ musculoskeletal health, we obtained grip strength data from two survey cycles, 2011–2012 and 2013–2014. The dataset with grip strength as an outcome is referred to as the muscle strength dataset. Considering the higher prevalence of osteoporosis occurring after the age of 50 and the limitations of the NHANES database, individuals aged 80 and over are topcoded at 80 years. Our study cohort comprised individuals aged between 50 and 80 years who had complete records of dietary intake, femoral neck BMD, and grip strength assessments. The intricate process of participant selection and inclusion is detailed in Figure S1, which provides a transparent overview of our rigorous methodology.

### 2.2. Dietary Intake Measures

Dietary data were meticulously collected through in-person 24 h recall interviews using the United States Department of Agriculture (USDA) Automated Multiple-Pass Method, a method known for its reliability and comprehensiveness. Our primary focus was on the first day’s dietary recall, a well-established and standardized approach that is widely used to obtain unbiased mean dietary intake estimates and is particularly applicable to studies involving large and diverse populations.

To ensure the precision of our analysis, we used the extensive resources of the USDA Food and Nutrient Database for Dietary Studies. This robust database facilitated the calculation of daily intakes for both macronutrients and micronutrients, providing a granular understanding of participants’ nutrient profiles. Given that water is an essential nutrient and constitutes the most abundant component in the human body, our study incorporated water, energy, protein, carbohydrate, and total fat into the macronutrient group. According to the collection of water intake data in the NHANES database, the included water intake included the total water drunk yesterday—including plain tap water, water from a drinking fountain, water from a water cooler, bottled water, and spring water. The mixed micronutrient intake encompassed all vitamins and minerals surveyed in the NHANES database, including vitamin A (VA), vitamin B6 (VB6), vitamin B12 (VB12), vitamin C (VC), VD, vitamin E (VE), vitamin K (VK), calcium, phosphorus, magnesium, iron, zinc, copper, sodium, potassium, and selenium.

### 2.3. Outcome Measures

In this study, we focused on two critical outcome variables, namely femoral neck BMD and grip strength, each of which plays a pivotal role in the assessment of musculoskeletal health. BMD, a fundamental measure of bone density, was meticulously assessed using dual-energy X-ray absorptiometry (DXA), a widely accepted gold-standard technique, using Hologic Discovery model A densitometers from Hologic, Inc. (Bedford, MA, USA). Concurrently, grip strength, an important indicator of upper extremity muscle strength, was quantified using Takei’s advanced digital handheld dynamometer (model T.K.K.5401). The rigorous methodology included a pretest and the collection of three measurements for each hand, separated by a brief 1 min rest interval to mitigate the effects of intra-thoracic pressure. Importantly, participants who were unable to complete single-handed testing were excluded from our study. For the subsequent analysis, we selected the maximum grip strength value derived from three consecutive trials for both the left and right hand.

### 2.4. Covariates

To control for the influence of potential confounding variables, such as socio-demographic characteristics and lifestyle factors, we integrated the following variables into our analysis. Socio-demographic factors included age (in years), body mass index (BMI) (categorized as normal or overweight), gender (categorized as male or female), education level (categorized as less than high school, high school or equivalent, or college or higher), race/ethnicity, marital status (categorized as married, never married, or widowed/divorced/separated), and poverty status (categorized as yes or no). We also included lifestyle behaviors, including smoking status (categorized as never, current, or former) and alcohol consumption (categorized as never, former, moderate, mild, or heavy).

## 3. Statistical Analyses

Statistical analyses were conducted using R version 4.2.2, with statistical significance defined as a *p*-value below 0.05. Continuous variables were presented as survey-weighted means and standard deviations (SDs), while categorical variables were represented as survey-weighted proportions. Student’s *t*-test and the chi-squared test were applied to assess differences in continuous and categorical variables, respectively.

### 3.1. Statistical Model 1: Multivariate Linear Regression Model

Our analysis began with a multivariate regression model examining the relationships between individual nutrient intakes and both BMD and grip strength. All multiple linear regression analyses used sample weights. Two models were constructed: a crude model with no covariate adjustments and Model 1, which was adjusted for age, gender, BMI, race, education level, smoking status, marital status, poverty status, and alcohol consumption. Linear regression results were presented as coefficients (β) with 95% confidence intervals (CI).

### 3.2. Statistical Model 2: The Restricted Cubic Spline (RCS) Regression Model

We then sought to visualize the dose–response relationships between individual nutrient intakes and both BMD and grip strength. To account for potential nonlinear associations, we used an unweighted RCS regression model and analysis of variance (ANOVA) plotted at three nodes (10th, 50th, 90th) because no RCS model was available for complex, multistage survey data.

### 3.3. Statistical Model 3: Weighted Quantile Sum (WQS) Regression Model

The WQS analysis is a multivariate approach that evaluates the combined effect of all predictor variables on the outcome using a weighted index. The relative weight of each variable is used to determine its contribution to the overall exponential effect [15].

In our subsequent analysis, we used the WQS regression model to examine the correlations between mixed macronutrient and micronutrient intake and BMD and grip strength. This model adjusted for several covariates, including age, gender, BMI, race, education level, smoking status, marital status, poverty status, and alcohol consumption, using a weighted quartile sum approach. Applicable to both linear and logistic regression, the WQS model included all measured nutrients and assigned weights to reflect their cumulative effect and relative contribution. WQS regression can be used to assess the dependency of an outcome on a weighted index, indicating coexposure to macronutrients and micronutrients.

### 3.4. Statistical Model 4: Quantile-Based g-Computation (QGC) Regression Model

Finally, the QGC regression model was used, which provides a novel approach that combines WQS reasoning with the flexibility of g-computation. This model uniquely accounts for mixture components with both positive and inverse associations, allowing for a comprehensive examination of nutrient combinations and their effects on health outcomes. This model also adjusted for age, gender, BMI, race, education level, smoking status, marital status, poverty status, and alcohol consumption. The weights of each nutrient were used to represent the proportion of positive or negative contributions to the mixture exposure, with the range of each exposure set to 0–1 and the positive or negative weights summed to 1.

## 4. Results

### 4.1. Characteristics of Participants

For the assessment of socio-demographic and behavioral characteristics, population-weighted means for each dataset are detailed in Table S1. The bone mass dataset consisted of 6340 individuals, including 3294 men, with a mean age of 63.20 years. The muscle strength dataset consisted of 2533 participants, including 1228 men, with a mean age of 63.60 years. Notably, men were significantly more likely to drink alcohol and smoke than women across all datasets (*p* < 0.001). In addition, men had higher mean femoral neck BMD and better grip strength than women, both of which were statistically significant (*p* < 0.001).

### 4.2. Association between Individual Nutrient Intake and Bone Mass and Muscle Strength

We first examined the linear relationships between individual nutrient intake and both bone mass and muscle strength. In the bone mass dataset, we observed positive correlations of macronutrients (such as protein and water) and several micronutrients (including VB12, VC, VD, phosphorus, magnesium, zinc, copper, sodium, potassium, and selenium) with BMD after adjusting for potential confounders (Table S2). Conversely, in the muscle strength dataset, only water, potassium, and selenium showed a positive association with grip strength.

Figures S2 and S3 illustrate the exposure–response relationships between these macronutrients and BMD and grip strength, respectively. These results suggest a linear relationship between the five macronutrients and both BMD and grip strength. However, our results from RCS regression analysis for micronutrients reveal a different pattern. Specifically, calcium (*p* for nonlinearity = 0.011) and sodium (*p* for nonlinearity = 0.016) both showed nonlinear associations with BMD (Figure S4). In addition, a nonlinear relationship was observed between calcium and grip strength (*p* for nonlinearity = 0.002), as shown in Figure S5.

### 4.3. Association between Mixed Dietary Intake and Bone Mass

Recognizing the limitations of examining individual nutrient intakes, we used the WQS and QGC regression models to assess the combined effects of mixed macronutrient and micronutrient intakes on BMD.

**Figure 1:**
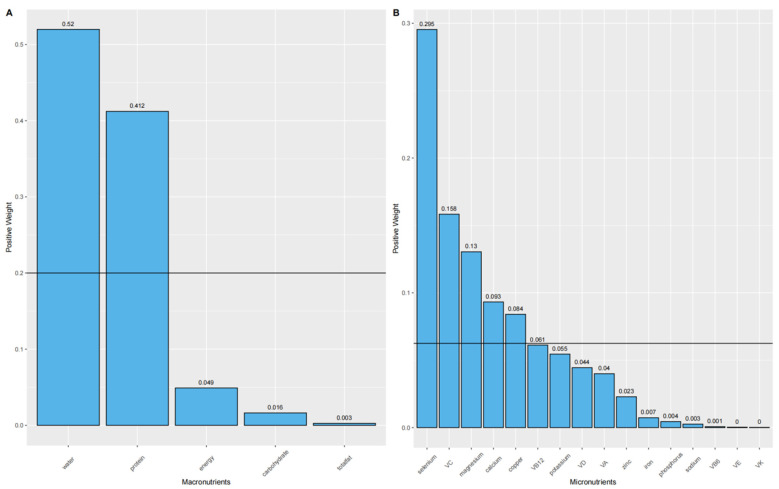
The WQS regression model estimated the weights of each macronutrient (**A**) and each micronutrient (**B**) associated with BMD. The sum of the weights of all nutrients in the figure was 1. The black solid line represented the average weight, 1/n, where n was the total number of nutrients considered in the analysis ((**A**): five; (**B**): sixteen).

Our initial analysis using the WQS model revealed a significant positive correlation between the WQS index of mixed macronutrient intake and BMD (β = 0.009, 95% CI:0.003, 0.014; *p* = 0.001). As shown in Figure 1A, this relationship was predominantly driven by water (weight = 0.52), followed by protein (weight = 0.416). In the case of mixed micronutrient intake, the WQS index also showed a significant positive association with BMD (β = 0.007, 95% CI:0.001, 0.012; *p* = 0.017). Selenium emerged as the most significant contributor (Figure 1B and Table 1), with VC (weight = 0.158), magnesium (weight = 0.13), calcium (weight = 0.093), and copper (weight = 0.084) also making substantial contributions among the sixteen micronutrients assessed (exceeding the horizontal line of 1/16).

**Figure 2:**
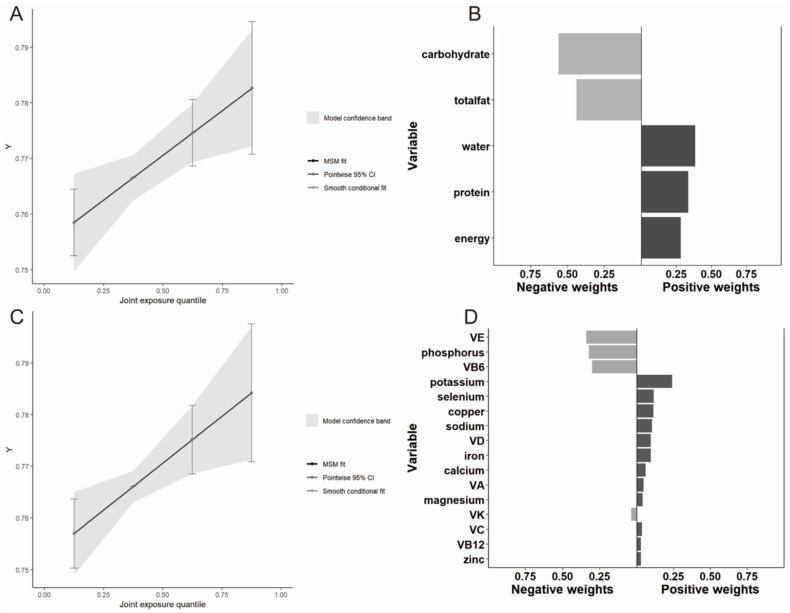
The proportion of positive and negative effects of dietary intake on BMD and the combined effects of mixed dietary intake to BMD in the QGC model. (**A**) The role of mixed macronutrients intake; (**B**) the proportion of the positive or negative effects for each macronutrient; (**C**) the role of mixed micronutrients intake; (**D**) the proportion of the positive or negative effects for each micronutrient.

Subsequent analysis using the QGC model yielded consistent results, confirming the beneficial effect of mixed dietary intakes of both macronutrients and micronutrients on BMD (Table 1). Figure 2 illustrates the distribution of nutrient weights in these associations. Notably, water was the most influential factor in the positive direction for mixed macronutrient intake, with protein also playing a significant role (Figure 2B). In the mixed micronutrient model, potassium had the highest weight, followed closely by selenium, copper, sodium, and VD (Figure 2D).

### 4.4. Association between Mixed Dietary Intake and Muscle Strength

**Figure 3:**
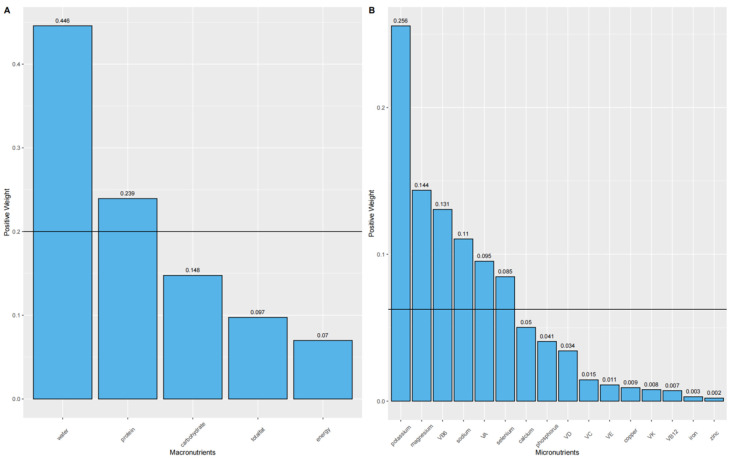
The WQS regression model estimated the weights of each macronutrient (**A**) and each micronutrient (**B**) associated with grip strength. The sum of the weights of all nutrients in the figure was 1. The black solid line represented the average weight, 1/n, where n was the total number of nutrients considered in the analysis ((**A**): five; (**B**): sixteen).

The results of the WQS regression model indicate a significant positive correlation between both mixed macronutrient and micronutrient intakes and grip strength (Table 1). Specifically, for mixed macronutrient intake, a one-quartile increase in the WQS index corresponded to a 0.670 unit increase in grip strength (β = 0.670, 95% CI: 0.225, 1.116; *p* = 0.003). Mirroring the pattern observed for BMD, water (weight = 0.446) and protein (weight = 0.239) were the most influential factors (Figure 3A). In the mixed micronutrient intake group, potassium (weight = 0.256) and magnesium (weight = 0.144) had the greatest effect, followed by VB6 (weight = 0.131), sodium (weight = 0.11), and VA (weight = 0.095) (Figure 3B).

**Figure 4:**
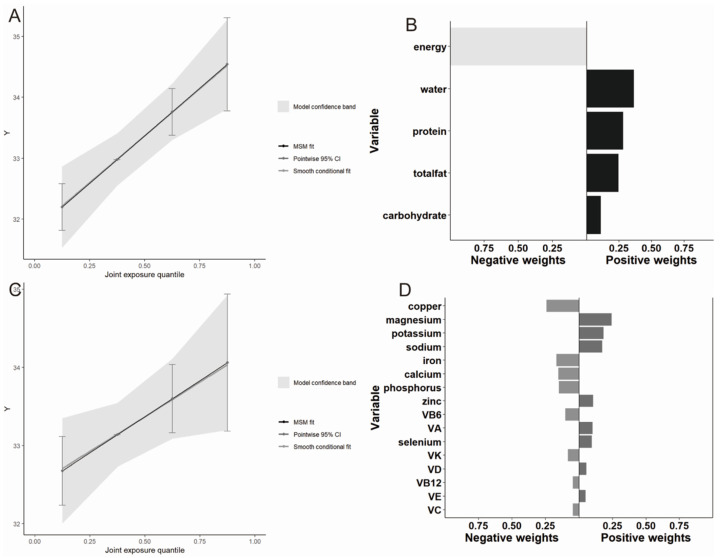
The proportion of positive and negative effects of dietary intake on grip strength and the combined effects of mixed dietary intake on grip strength in the QGC model. (**A**) The role of mixed macronutrients intake; (**B**) the proportion of the positive or negative effects for each macronutrient; (**C**) the role of mixed micronutrient intake; (**D**) the proportion of the positive or negative effects for each micronutrient.

Analysis using the QGC model further supported these findings. Mixed macronutrient intake was positively associated with grip strength (β = 0.783, 95% CI:0.400, 1.165; *p* < 0.001). Similarly, the overall effect of mixed micronutrient intake was positively associated with grip strength (β = 0.462, 95% CI:0.023, 0.090; *p* = 0.039). Consistent with the WQS model, water and protein had the most significant positive contributions in the mixed macronutrient group, whereas magnesium, potassium, and sodium were predominant in the mixed micronutrient group (Figure 4).

## 5. Discussion

In this study, we used multiple statistical approaches to rigorously evaluate the association of individual and combined dietary intakes on bone and muscle. Our analyses revealed that the majority of individual nutrients were positively correlated with BMD and grip strength, highlighting the importance of different dietary components in musculoskeletal health. Significantly, the use of both WQS and QGC regression models revealed that the combined intake of macronutrients and micronutrients had a robust positive association with BMD and grip strength. This comprehensive approach underscores the synergistic effect of balanced dietary intake over isolated key nutrient supplementation. Notably, our findings reveal that protein and water were consistently the primary contributors to mixed macronutrient intake for both BMD and grip strength. Among the micronutrients, selenium was particularly influential in positively correlating with BMD, while potassium and magnesium were predominant in positively influencing grip strength. These findings are not only consistent with but significantly extend current nutritional science, suggesting that a holistic dietary approach is critical for optimal musculoskeletal health, particularly in older adults facing the dual challenges of osteoporosis and sarcopenia.

The musculoskeletal system, consisting of bone, muscle, and joints, naturally undergoes gradual tissue loss and degeneration, a consequence of lifelong mechanical and biological stress [16]. This relentless progression leads to various age-related diseases, most notably osteoporosis and sarcopenia. These conditions, which predominantly affect the elderly population, not only impair functional ability but also increase the risk of associated comorbidities [17]. Given the profound interconnectivity between muscle and bone, age-related deterioration of one will inevitably affect the functionality of the other [18,19]. In recognition of this, our study emphasizes the interdependent nature of bone and muscle health and highlights the need for a unified approach to address the complexities of musculoskeletal aging. Our findings suggest that a comprehensive balanced dietary strategy may be critical in mitigating the deleterious effects of aging on these vital tissues.

The relationship between individual nutrient intake and bone and muscle health is well established in the extant literature. Consistent with our findings, previous studies have shown that older adults may have a diminished metabolic response to protein, necessitating increased protein intake to support bone matrix collagen and prevent loss of muscle mass and strength [20,21]. Increasing protein intake may increase serum levels of insulin-like growth factor 1 (IGF-1). IGF-1 is a key regulator of bone metabolism and acts as a systemic and local modulator of osteoblast function. It promotes bone remodeling by stimulating both bone resorption and bone formation [22]. As older adults often experience a decreased sense of thirst, it is critical for them to maintain adequate water intake for optimal bone and muscle health [23]. Adequate water intake helps maintain the balance of intracellular and extracellular fluids, promotes smooth blood circulation, enhances the delivery efficiency of nutrients and hormones, supports normal nervous system function, and improves the excitability and conduction speed of motor neurons. This, in turn, increases the contractile capacity and strength of muscle fibers [24,25].

Additionally, the role of micronutrients in supporting bone and muscle health has been supported by previous research. Studies by Feskanich et al. and Dzik et al. highlight the efficacy of calcium and VD in reducing bone loss and improving muscle strength and function, respectively [4,26,27]. VD may enhance the beneficial effects of Ca on muscle by improving its bioavailability and potentiating its anabolic effects [28]. Our study extends this understanding by highlighting the importance of selenium, potassium, and magnesium. Selenium’s role in redox homeostasis and its antioxidant properties are essential for bone health [29,30]. Magnesium is essential for maintaining the structure and function of bone cells. Low magnesium levels can lead to hypoparathyroidism, resulting in hypocalcemia, which stimulates parathyroid hormone secretion and promotes bone resorption, thereby disrupting the balance of bone metabolism. Dietary potassium is also essential for musculoskeletal health, and severe potassium deficiency often results in muscle weakness and paralysis [31].

Our research fills a gap in understanding the impact of a comprehensive, balanced dietary intake on bone and muscle health. Given the physiological changes in older adults, such as reduced chewing and digestive ability and altered appetite, a comprehensive approach to nutrition is essential [13,32]. Unlike previous studies that focused on isolated key nutrients or specific groups such as water-soluble vitamins [33], our study examines broader dietary habits using the NHANES dataset. By not isolating bone from muscle, our research reflects the interconnected nature of the musculoskeletal system and provides a more holistic view of the role of diet in maintaining musculoskeletal health. This approach is particularly relevant given the typical dietary patterns of older adults and underscores the importance of a balanced intake of energy, macronutrients, and micronutrients.

Although our study provides valuable insights, it is important to acknowledge certain limitations. First, the cross-sectional design of the study limits the ability to establish causal relationships between dietary intake and musculoskeletal health outcomes. Secondly, the dietary data were self-reported, which may introduce measurement errors and recall bias. Additionally, the exclusion of participants with incomplete data might introduce selection bias, potentially impacting the generalizability and interpretation of our findings. These constraints underscore the necessity for future longitudinal studies to corroborate and expand upon our results. Third, although we accounted for several factors affecting BMD in our analysis, we cannot exclude the influence of genetic factors, gene–diet interactions, and gut microbiota composition on BMD. What is more, water intake may serve as a surrogate for physical activity in the analyses. However, in the existing database, due to the diverse categorization of physical activities without a standardized criterion, no further adjustments have been made. Finally, since the NHANES dataset is limited to the U.S. population, the generalizability of the findings to populations in other regions may be limited. Future research should focus on longitudinal studies to better understand the causal relationships between nutrient intake and musculoskeletal health. In addition, the inclusion of objective measures of dietary intake such as biomarkers could help reduce the measurement error associated with self-reported data. Further research is needed to validate these findings in diverse populations.

## 6. Conclusions

In our analysis of the NHANES dataset, we reveal the association of combined macro- and micronutrient intakes in improving musculoskeletal health in older adults. By demonstrating the synergistic effects of nutrients such as protein, water, selenium, potassium, and magnesium on bone mineral density and grip strength, our findings challenge traditional isolated key nutrient supplementation approaches. This research underscores the need for holistic nutritional strategies in the management of nutrition in the elderly and provides a new direction for future dietary guidelines and interventions. While our cross-sectional study design requires cautious interpretation, it paves the way for future research to explore the diverse nutritional needs of aging populations. This work highlights the transformative potential of comprehensive nutrition in counteracting age-related musculoskeletal decline and invites further exploration in this important area.

## Figures and Tables

**Table 1:** Association between mixed dietary intake and BMD and grip strength via WQS regression and QGC models.

Model	Outcomes	Mixed Dietary Patterns	β (95% CI)	*p*
WQS	BMD	Macronutrients	0.009 (0.003, 0.014)	0.001
		Micronutrients	0.007 (0.001, 0.012)	0.017
	Grip Strength	Macronutrients	0.670 (0.225, 1.116)	0.003
		Micronutrients	0.442 (0.041, 0.842)	0.031
QGC	BMD	Macronutrients	0.008 (0.002, 0.014)	0.008
		Micronutrients	0.009 (0.002, 0.016)	0.008
	Grip Strength	Macronutrients	0.783 (0.400, 1.165)	<0.001
		Micronutrients	0.462 (0.023, 0.90)	0.039

Models adjusted for age, gender, BMI, race, education level, smoking status, marital status, poverty status, and alcohol consumption.

## Data Availability

The datasets used and/or analyzed during the current study are available from https://www.cdc.gov/nchs/nhanes/nhanes_questionnaires.htm (accessed on 1 December 2023).

## Supplementary Materials

The following supporting information can be downloaded at https://www.mdpi.com/article/10.3390/nu16111640/s1. Table S1. Characteristics of the participants; Table S2. Association between individual nutrient intake and bone mass and muscle strength; Figure S1. Flowchart of sample selection; Figure S2. Restricted cubic spline (RCS) analysis with multivariate-adjusted associations between macronutrients intake (A: energy; B: protein; C: water; D: carbohydrate; E: total fat) and BMD. Models are adjusted for age, gender, BMI, race, education level, smoking status, marital status, poverty status, and alcohol consumption; Figure S3. Restricted cubic spline (RCS) analysis with multivariate-adjusted associations between macronutrients intake (A: energy; B: protein; C: water; D: carbohydrate; E: total fat) and grip strength. Models are adjusted for age, gender, BMI, race, education level, smoking status, marital status, poverty status, and alcohol consumption; Figure S4. Restricted cubic spline (RCS) analysis with multivariate-adjusted associations between micronutrients intake (A: VA; B: VB6; C: VB12; D: VC; E: VD; F: VE; G: VK; H: calcium; I: phosphorous J: magnesium; K: iron; L: zinc; M: copper; N: sodium; O: potassium; P: selenium) and BMD. Models are adjusted for age, gender, BMI, race, education level, smoking status, marital status, poverty status, and alcohol consumption; Figure S5. Restricted cubic spline (RCS) analysis with multivariate-adjusted associations between micronutrients intake (A: VA; B: VB6; C: VB12; D: VC; E: VD; F: VE; G: VK; H: calcium; I: phosphorous J: magnesium; K: iron; L: zinc; M: copper; N: sodium; O: potassium; P: selenium) and grip strength. Models are adjusted for age, gender, BMI, race, education level, smoking status, marital status, poverty status, and alcohol consumption.
