# Supplementary material for: Synergistic Effects of Nutrients on Musculoskeletal Health in Gerontology: Understanding the Combined Impact of Macronutrients and Micronutrients
Source: Nutrients. 2024 May 27;16(11):1640. doi: 10.3390/nu16111640 (PMC11174030; doi:10.3390/nu16111640)
Supplement: Supplementary file 1 [file nutrients-16-01640-s001.zip › nutrients-2976990-supplementary.pdf]

Table S1. Characteristics of the participants.

Variable	Bone mass dataset [a]				Muscle strength dataset [b]			
	Total(n=6340)	Men(n=3294)	Women(n=3046)	*P*	Total(n=2533)	Men(n=1228)	Women(n=1305)	*P*
Age [yrs, mean(SD)]	63.197(0.199)	62.936(0.255)	63.436(0.229)	0.074	63.601(0.293)	63.403(0.390)	63.770(0.332)	0.392
BMI [n (%)]				0.009				0.308
Normal	1559(24.59)	771(21.271)	788(25.972)		642(25.345)	323(24.121)	319(26.552)	
Overweight	4781(75.41)	2523(78.729)	2258(74.028)		1891(74.655)	905(75.879)	986(73.448)	
Race/ethnicity [n (%)]				0.368				0.143
Non-Hispanic White	3008(47.445)	1554(74.329)	1454(75.689)		1202(47.454)	562(75.754)	640(76.061)	
Mexican American	834(13.155)	414(5.273)	420(5.332)		237(9.356)	128(4.818)	109(3.946)	
Non-Hispanic Black	1348(21.262)	735(10.006)	613(8.743)		615(24.28)	303(9.650)	312(10.187)	
Other Hispanic	607(9.574)	315(4.330)	292(3.759)		247(9.751)	108(3.607)	139(4.971)	
Other Race - Including Multi-Racial	543(8.565)	276(6.061)	267(6.476)		232(9.159)	127(6.171)	105(4.835)	
Smoke [n (%)]				< 0.001				0.001
Former	2163(34.117)	1393(40.510)	770(26.901)		1232(48.638)	469(40.353)	763(54.065)	
Never	3153(49.732)	1279(43.340)	1874(59.016)		436(17.213)	237(18.077)	199(16.225)	
Now	1024(16.151)	622(16.151)	402(14.083)		865(34.149)	522(41.570)	343(29.709)	
Drinking [n (%)]				< 0.001				< 0.001
Former	1074(16.94)	595(14.311)	479(12.985)		642(25.345)	335(21.315)	307(19.105)	
Heavy	735(11.593)	480(14.858)	255(8.577)		270(10.659)	170(15.719)	100(9.318)	
Never	917(14.464)	242(6.379)	675(16.803)		412(16.265)	99(6.310)	313(18.530)	
Moderate	875(13.801)	344(11.968)	531(21.576)		923(36.439)	512(46.400)	411(36.965)	
Mild	2739(43.202)	1633(52.485)	1106(40.059)		286(11.291)	112(10.255)	174(16.081)	
Education level [n (%)]				0.841				0.075
High school or equivalent	690(10.883)	353(10.228)	337(10.606)		608(24.003)	280(21.045)	328(25.296)	
Lower than high school	947(14.937)	511(8.648)	436(8.302)		1263(49.862)	602(60.320)	661(58.336)	
College or above	4703(74.18)	2430(81.124)	2273(81.093)		662(26.135)	346(18.635)	316(16.368)	
Marital level [n (%)]				< 0.001				< 0.001
Married	2912(45.931)	1741(58.626)	1171(46.546)		1379(54.441)	806(69.842)	573(52.997)	
Widowed/Divorced/Separated	3044(48.013)	1351(35.138)	1693(48.959)		967(38.176)	334(23.306)	633(41.311)	
Never married	384(6.057)	202(6.236)	182(4.495)		187(7.383)	88(6.852)	99(5.692)	
Poverty status [n (%)]				0.799				0.228
Yes	1029(16.23)	533(9.388)	496(9.551)		490(19.345)	216(11.663)	274(13.581)	
No	5311(83.77)	2761(90.612)	2550(90.449)		2043(80.655)	1012(88.337)	1031(86.419)	
BMD [gm/cm^2 , mean(SD)]	0.762(0.003)	0.810(0.003)	0.718(0.003)	< 0.001				
Grip strength [kg, mean(SD)]					33.911(0.332)	42.540(0.457)	26.512(0.223)	< 0.001

Data are mean (SE) or N (%)

[a] five cycles dietary Day 1 sample weights (2007-2008, 2009-2010, 2013-2014, 2017-2018, 2019-2020)

[b] two cycles dietary Day 1 sample weights (2011-2012, 2013-2014

Table S2. Association between individual nutrient intake and bone mass and muscle strength.

		Bone mass dataset [a]				Muscle strength dataset [b]			
		Crude model		Model1		Crude model		Model1	
	character	β (95% CI)	*P*	β (95% CI)	*P*	β (95% CI)	*P*	β (95% CI)	*P*
Macronutrient Group	energy	0.162(0.123,0.200)	<0.0001	0.015(-0.028, 0.058)	0.496	25.124(21.355,28.892)	<0.0001	1.262(-1.072, 3.595)	0.264
	protein	0.184(0.156,0.212)	<0.0001	0.061(0.029, 0.094)	<0.001	21.915(17.968,25.863)	<0.0001	1.721(-0.680, 4.123)	0.146
	carbohydrate	0.099(0.060,0.139)	<0.0001	-0.005(-0.048, 0.037)	0.803	17.448(14.226,20.670)	<0.0001	0.358(-2.020, 2.735)	0.750
	Total fat	0.121(0.091,0.150)	<0.0001	0.01(-0.022, 0.043)	0.522	16.681(12.610,20.752)	<0.0001	1.308(-0.839, 3.455)	0.211
	water	0.035(0.013,0.056)	0.002	0.027(0.005, 0.049)	0.016	-0.541(-3.487,2.405)	0.711	1.179(0.134, 2.224)	0.030
Micronutrient Group	VA	0.011(-0.015,0.038)	0.402	0.02(-0.006, 0.047)	0.131	2.049(-1.701,5.798)	0.274	0.874(-1.263, 3.010)	0.393
	VB6	0.097(0.065,0.129)	<0.0001	0.023(-0.010, 0.056)	0.168	15.699(13.062,18.337)	<0.0001	0.504(-1.184, 2.192)	0.530
	VB12	0.086(0.059,0.113)	<0.0001	0.038(0.006, 0.070)	0.019	9.716(5.447,13.985)	<0.0001	1.062(-1.572, 3.696)	0.399
	VC	0.024(0.001,0.046)	0.039	0.021(0.000, 0.042)	0.048	3.67(-0.024,7.364)	0.051	0.941(-1.452, 3.334)	0.411
	VD	0.032(0.010,0.055)	0.006	0.025(0.005, 0.046)	0.017	2.919(-0.487,6.325)	0.090	1.387(-0.995, 3.769)	0.231
	VE	0.052(0.022,0.082)	<0.001	0.001(-0.032, 0.034)	0.948	11.114(7.605,14.623)	<0.0001	0.951(-1.023, 2.926)	0.317
	VK	0.02(-0.003,0.043)	0.081	0.009(-0.011, 0.029)	0.362	3.602(0.140,7.063)	0.042	1.049(-0.691, 2.790)	0.215
	calcium	0.068(0.042,0.095)	<0.0001	0.02(-0.009, 0.048)	0.173	7.796(4.324,11.268)	<0.0001	1.076(-1.294, 3.445)	0.345
	phosphorus	0.157(0.128,0.187)	<0.0001	0.055(0.019, 0.090)	0.003	19.339(15.522,23.155)	<0.0001	1.044(-1.475, 3.562)	0.387
	magnesium	0.131(0.094,0.168)	<0.0001	0.052(0.013, 0.092)	0.011	17.572(13.541,21.602)	<0.0001	1.381(-0.658, 3.420)	0.167
	iron	0.098(0.066,0.130)	<0.0001	0.029(-0.005, 0.064)	0.096	11.9(9.332,14.468)	<0.0001	-0.307(-2.253, 1.640)	0.739
	zinc	0.136(0.108,0.164)	<0.0001	0.05(0.019, 0.081)	0.002	15.891(12.015,19.768)	<0.0001	0.957(-1.103, 3.017)	0.334
	copper	0.109(0.078,0.140)	<0.0001	0.049(0.017, 0.080)	0.003	12.009(8.023,15.995)	<0.0001	0.255(-1.863, 2.374)	0.799
	sodium	0.152(0.121,0.182)	<0.0001	0.041(0.009, 0.072)	0.012	21.339(17.284,25.394)	<0.0001	1.171(-1.261, 3.603)	0.317
	potassium	0.118(0.078,0.159)	<0.0001	0.051(0.008, 0.093)	0.020	20.008(16.760,23.257)	<0.0001	2.039(0.411, 3.667)	0.018
	selenium	0.152(0.124,0.180)	<0.0001	0.042(0.012, 0.073)	0.007	19.873(16.426,23.320)	<0.0001	2.35(0.223, 4.478)	0.033

[a] five cycles dietary Day 1 sample weights (2007-2008, 2009-2010, 2013-2014, 2017-2018, 2019-2020)

[b] two cycles dietary Day 1 sample weights (2011-2012, 2013-2014)

Crude model: no covariate was adjusted.

Model1: age, gender, BMI, race, education level, smoking status, marital status, poverty status, and alcohol consumption were adjusted.

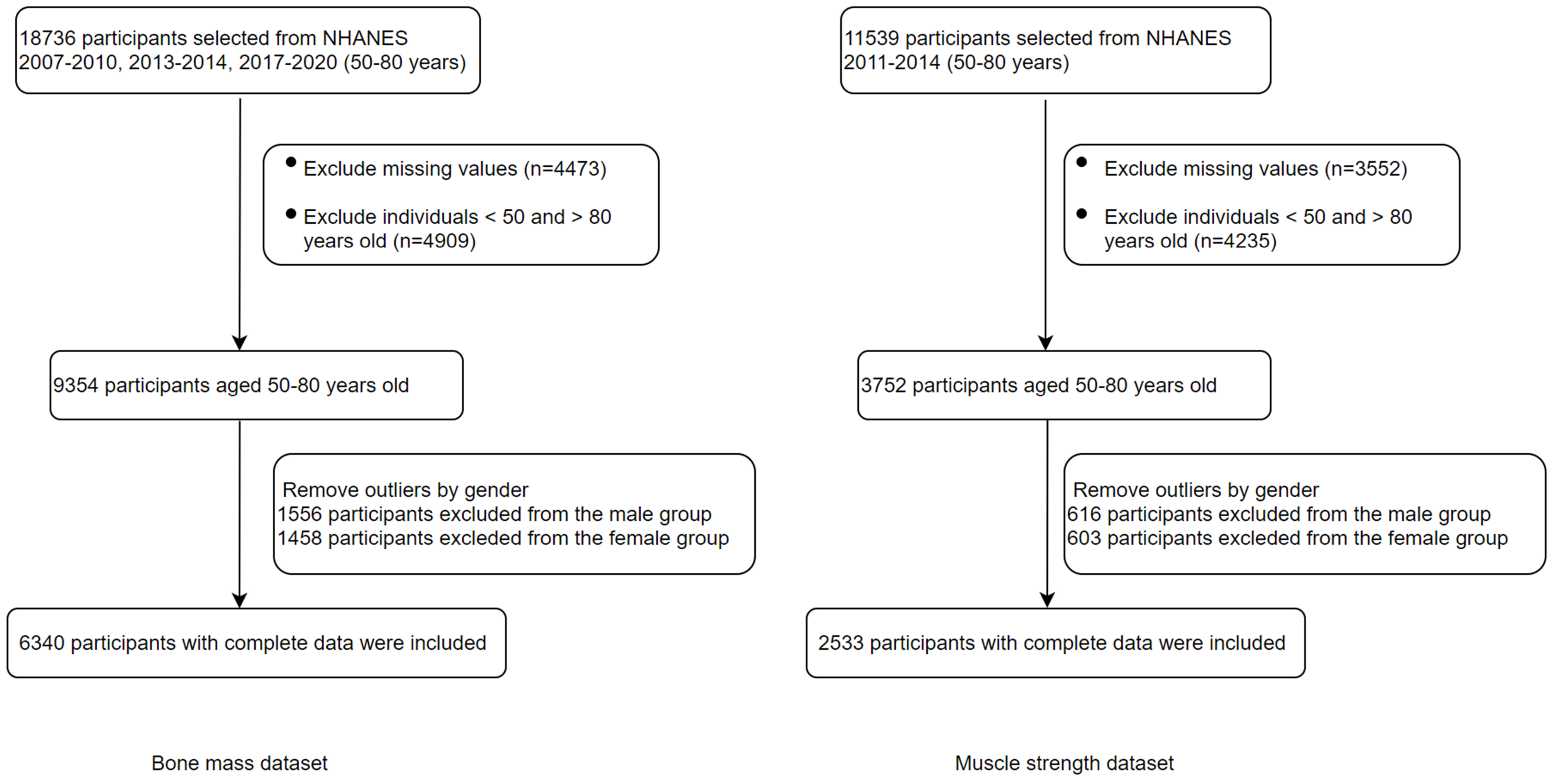

Figure S1. Flowchart of sample selection.

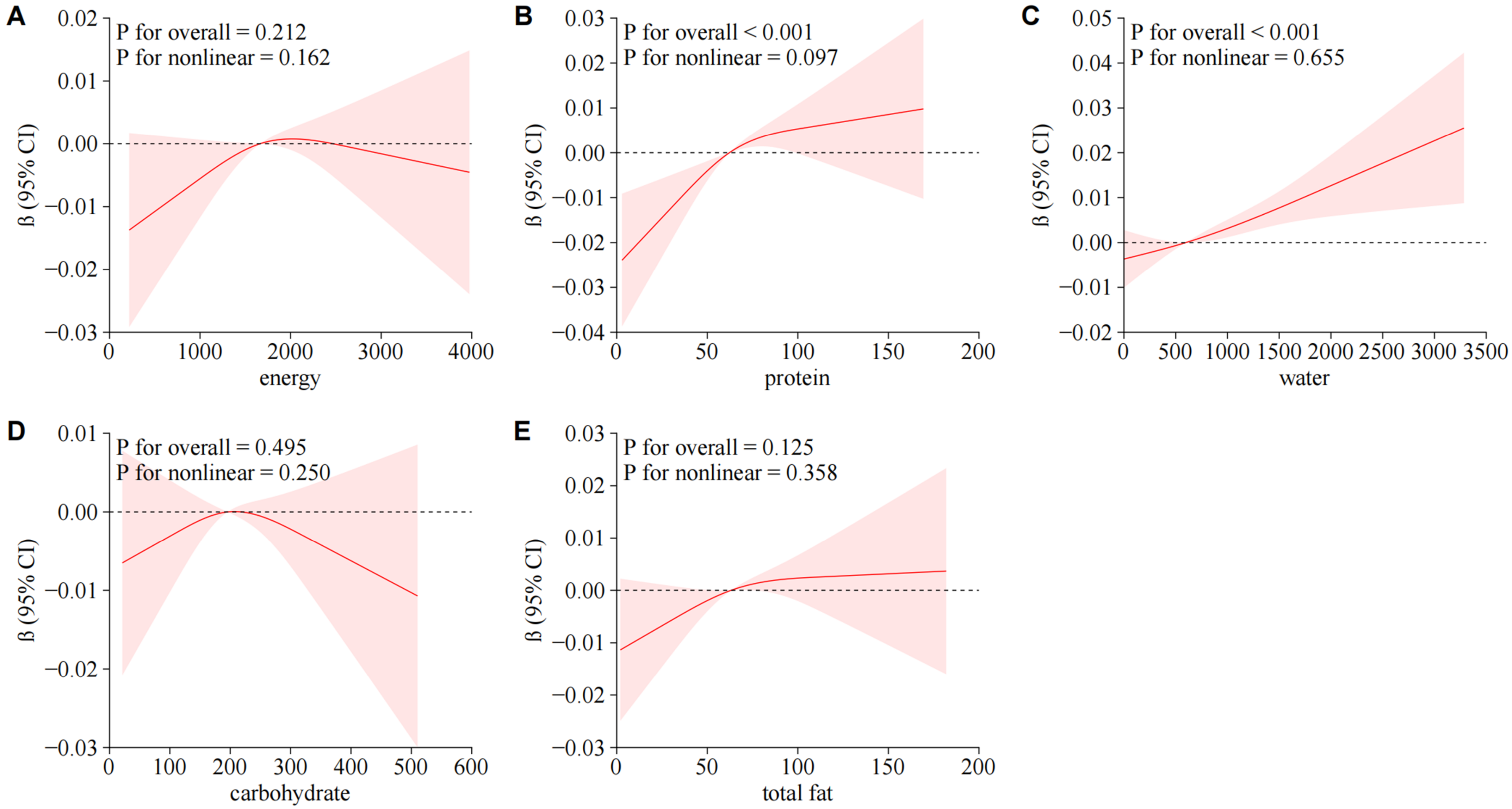

Figure S2. Restricted cubic spline (RCS) analysis with multivariate-adjusted associations between macronutrients intake (A: energy; B: protein; C: water; D: carbohydrate; E: total fat) and BMD. Models are adjusted for age, gender, BMI, race, education level, smoking status, marital status, poverty status, and alcohol consumption.

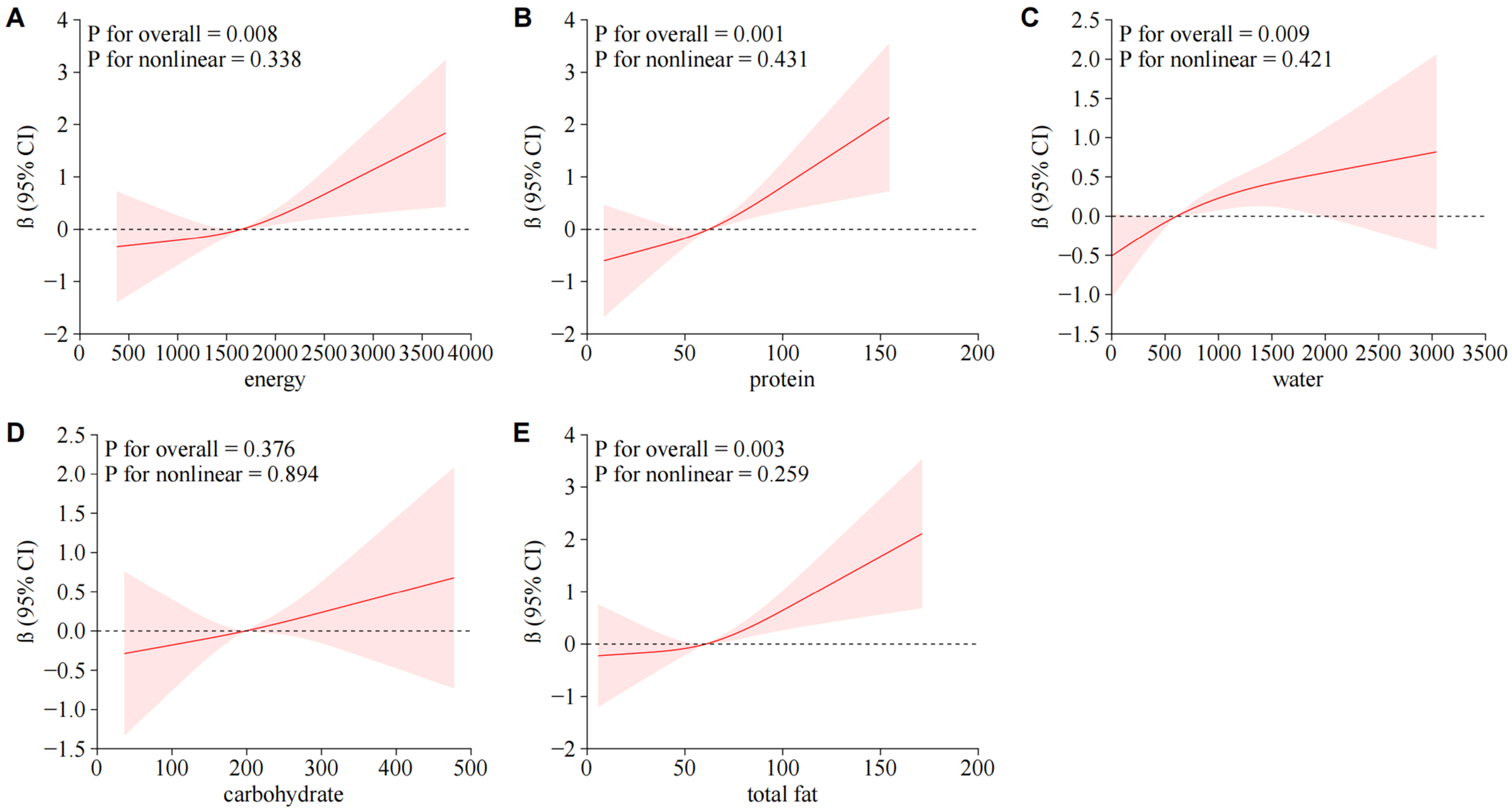

Figure S3. Restricted cubic spline (RCS) analysis with multivariate-adjusted associations between macronutrients intake (A: energy; B: protein; C: water; D: carbohydrate; E: total fat) and grip strength. Models are adjusted for age, gender, BMI, race, education level, smoking status, marital status, poverty status, and alcohol consumption.

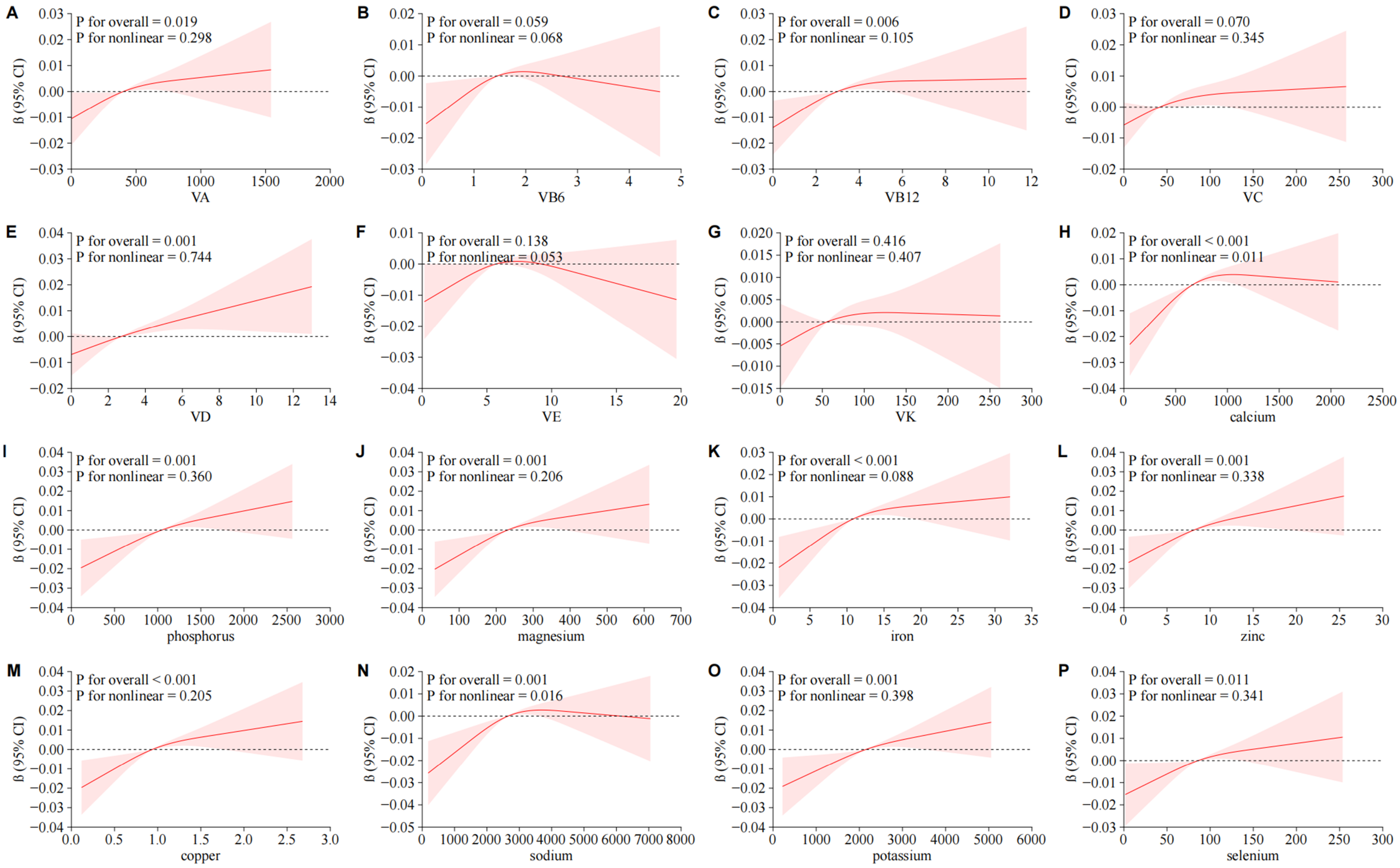

Figure S4. Restricted cubic spline (RCS) analysis with multivariate-adjusted associations between micronutrients intake (A: VA; B: VB6; C: VB12; D: VC; E: VD; F: VE; G: VK; H: calcium; I: phosphorous J: magnesium; K: iron; L: zinc; M: copper; N: sodium; O: potassium; P: selenium) and BMD. Models are adjusted for age, gender, BMI, race, education level, smoking status, marital status, poverty status, and alcohol consumption.

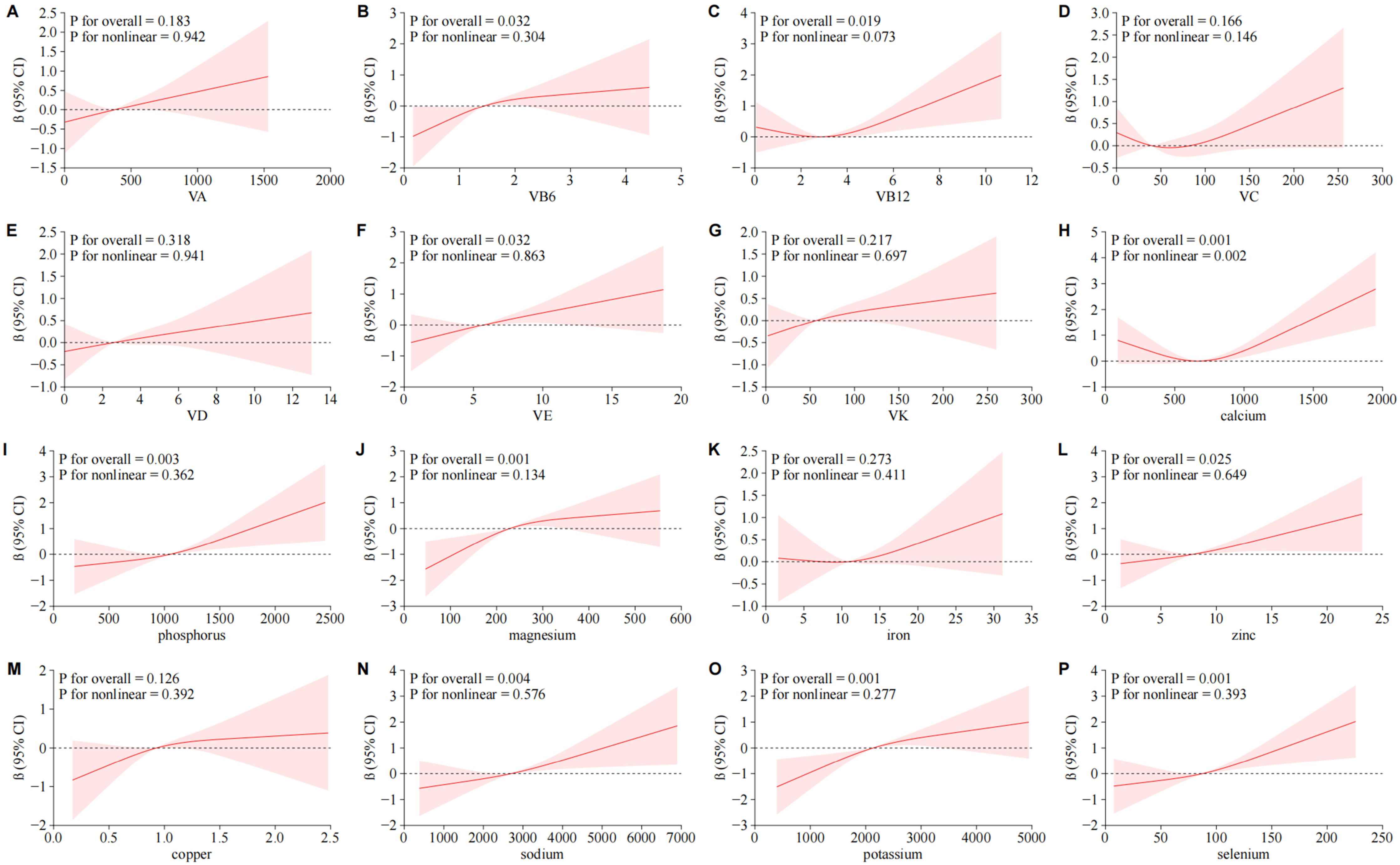

Figure S5. Restricted cubic spline (RCS) analysis with multivariate-adjusted associations between micronutrients intake (A: VA; B: VB6; C: VB12; D: VC; E: VD; F: VE; G: VK; H: calcium; I: phosphorous J: magnesium; K: iron; L: zinc; M: copper; N: sodium; O: potassium; P: selenium) and grip strength. Models are adjusted for age, gender, BMI, race, education level, smoking status, marital status, poverty status, and alcohol consumption.
